# MicroRNA‐488 inhibits endometrial glandular epithelial cell proliferation, migration, and invasion in endometriosis mice via Wnt by inhibiting FZD7

**DOI:** 10.1111/jcmm.14078

**Published:** 2019-02-07

**Authors:** Hui Zhu, Xi‐Xia Cao, Juan Liu, Hua Hua

**Affiliations:** ^1^ Department of Reproductive Health Nanjing Maternity and Child Health Care Hospital, The Affiliated Obstetrics and Gynecology Hospital of Nanjing Medical University Nanjing P. R. China; ^2^ Department of Gynecology Nanjing Maternity and Child Health Care Hospital, The Affiliated Obstetrics and Gynecology Hospital of Nanjing Medical University Nanjing P. R. China

**Keywords:** endometrial glandular epithelial cells, Endometriosis, FZD7, microRNA‐488, Wnt signalling pathway

## Abstract

Endometriosis is a chronic inflammatory syndrome and nearly 6%‐10% of women are affected by it during the reproductive period. Previous studies have proved that microRNAs (miRNAs) are implicated in the pathogenesis of ovarian endometriosis. In this study, we aimed to investigate that restored miR‐488 would effectively inhibit the development of endometriosis. The microarray‐based data analysis was performed to screen endometriosis‐related differentially expressed genes (DEGs). The mouse model in endometriosis syndrome was established by being subcutaneously injected with Estradiol benzoate, and the ectopic endometrial tissues and normal endometrial tissues were collected. Additionally, the endometrial glandular epithelial cells were extracted from the endometrial glandular epithelial tissues from normal and endometriosis mice. In order to examine the role of miR‐488 in mice with endometriosis, we measured miR‐488 expression and expression levels of Frizzled‐7 (FZD7), cyclinD1, β‐catenin, and c‐Myc in vivo and in vitro. Finally, we detected the effect of miR‐488 on cell proliferation, apoptosis, migration and invasion in vitro. FZD7 was upregulated in human endometriosis. The data showed higher expression levels of FZD7, β‐catenin, c‐Myc and cyclinD1, and lower miR‐488 expression in mouse endometrial tissues. FZD7 was the target gene of miR‐488. Furthermore, elevated miR‐488 in isolated mouse endometrial glandular endometrial cells inhibited FZD7, the translocation of β‐catenin to nucleus, the activation of Wnt pathway, and the cell proliferation, migration and invasion. Collectively, these findings indicated that up‐regulated miR‐488 may reduce the proliferation, migration and invasion of endometrial glandular epithelial cells through inhibiting the activation of Wnt pathway by down‐regulating FZD7.

## 1 INTRODUCTION

Endometriosis is a chronic inflammation disease characterized by the allotopic endometrial glands and stroma, which appear outside the uterine cavity.^1^ The pathogenesis of endometriosis is still largely unknown, and a study has provided evidence for the possibility that the progression of endometriosis may be attributed to inflammation, oxidative stress and immunological dysfunctions.^2^ The well‐known symptoms of endometriosis include dyschezia, deep dyspareunia, dysuria and dysmenorrhoea.^3^ The amount of women with endometriosis during reproductive years reaches 10%, and those who have pelvic or infertility are more likely to suffer from endometriosis.^4^ Although, a previous report indicated that the prevalence of endometriosis had decreased in a certain degree, the incidence of endometriosis was higher than several decades ago.^5^ A great deal of health‐care resources and persistent therapy are needed for the patients with endometriosis, since once the current medical therapies are discontinued, the alleviative symptoms are likely to recur and extra interventions are required.^6^ It has been indicated that gene therapy holds significant promise in the endometriosis treatment for patients who have very limited treatment options at present.^7^

MicroRNAs (miRNAs) are a group of endogenous small non‐coding RNAs that regulate translation of various target mRNAs, involving in most cellular and developmental processes as a novel and potent gene expression regulator.^8^ Recent study has revealed that an abnormal miRNA expression profile is responsible for the endometriosis by promoting the implantation of endometrial cells at aberrant sites through high angiogenic and proteolytic activities.^9^ miR‐488 is a kind of miRNA, which has been demonstrated to play positive role in ovarian cancer by regulating the mitochondrial function.^10^ Also, the potential role of miR‐488 as a tumour suppressor by inhibiting cell proliferation, cell migration, colony information, and the cell cycle in non‐small‐cell lung cancer and gastric cancer.^11^ Besides, it has been suggested by some researchers that the activation of Wnt/β‐catenin signalling pathway can be a potential mechanism under the fibrosis in endometriosis.^5^ Frizzled‐7 (FZD7), a member of the Frizzled family of transmembrane proteins, is a Wnt receptor that can activate the canonical and/or the non‐canonical Wnt signalling pathways.^12^ It has been demonstrated that the overexpressed FZD7 can contribute to the proliferation and growth of gliomas cells, contrarily, the low expression of FZD7 enormously inhibits the proliferation ability of gliomas cells.^13^ In this study, we hypothesised that the miR‐488 can regulate the proliferation, migration, and invasion of endometrial glandular epithelial cells through the Wnt signalling pathway by targeting FZD7, and we aim to explore new novel therapeutic targets for endometriosis.

## 2 MATERIAL AND METHODS

### 2.1 Ethics statement

This study was carried out in strict accordance with the recommendations in the Guide for the Care and Use of Laboratory Animals of the National Institutes of Health. The protocol was approved by the Institutional Animal Care and Use Committee of the Affiliated Obstetrics and Gynecology Hospital of Nanjing Medical University, Nanjing Maternity and Child Health Care Hospital.

### 2.2 Microarray‐based data analysis

NCBI is a public platform for storing gene expression datasets, original sequences and records. By retrieving endometriosis microarrays, we found the GSE5108 and GSE23339 chips and downloaded the data from Gene Expression Omnibus (GEO). It was found that GSE5108 included 11 endometriosis samples and 11 normal control samples, and GSE23339 included 10 endometriosis samples and nine normal control samples. Next, Bioconductor‐based “limma” package in r language was employed to select important differentially expressed genes (DEGs) with the empirical Bayes method. Finally, the DEGs were annotated using the “annotate” package. *P* < 0.05 was considered statistically significant. The miRNA target prediction websites (http://www.targetscan.org/vert_71/, http://www.mirdb.org/, http://diana.imis.athena-innovation.gr/DianaTools/index.php?r=microT_CDS/index) were used to predicate the miRNAs that regulated candidate gene FZD7, and the prediction results were then intersected.

### 2.3 Model establishment

A total of 70 healthy sexually‐mature specific‐pathogen‐free female mice of Institute of Cancer Research (ICR) weighting between 20 and 25 g with 6‐8 weeks of age were purchased from Shanghai Sippr BK laboratory animal Co., Ltd. (Shanghai, China). The mice were checked in fixed time every day for a week before the injection, and were performed with the ovariectomy to control the differences of oestrogen induced by different mice. The normal mice were chosen as study subjects, and they were subcutaneously injected with Estradiol benzoate (E2, 0.1 mg) in the first 3 days and anaesthetised by intraperitoneal injection of 1% pentobarbital sodium (40 mg/kg). One to two mice were chosen as donors, and their uterus were taken, washed with sterile D‐Hank's solution for several times to remove the blood clot and mucus, and placed in the Dulbecco's modified Eagle's medium (DMEM) and Ham's nutrient mixture F‐12 at room temperature. The endometrium was separated and the myometrium were removed in the culture dish containing normal saline. The endometrial tissues were cut into 2‐3 mm^3^ pieces for the operation. The normal mice were anaesthetised and fixed on the operation panel, the abdomen was disinfected and a 5 mm‐incision on the abdomen skin was made by using an ophthalmic scissors. The subcutaneous tissues were separated and implanted with two prepared 2‐3 mm^3^ endometrial tissues in the two sides of ventrimeson, respectively (no suture), and lastly the incision was sutured. After disinfection, the mice were placed in the cages. The emergence of subcutaneous node of endometriosis indicated the success of model establishment.^14^ The review was conducted 3 weeks after model establishment, the skin of model mice was opened as the previous operation to observe the growth and survival of implants. The criteria for the success of model establishment observed by naked eyes^15^ were as follows: the volume of implants increased, the implants were transparent nodositas and cystiform, the supernatant liquid accumulated, the implants were soft, covered by connective tissue, and blood vessels were formed.

### 2.4 Hematoxylin eosin staining

A total of 30 mice were sacrificed by air embolism method 6 weeks after model establishment. The uterus was removed in sterile environment, and the endometrial tissues were taken under dissecting microscope (2354353, Olympus Optical Co., Ltd., Tokyo, Japan). Part of tissue was taken and fixed in 4% formaldehyde, embedded in paraffin and cut into 5‐8 μm‐thick sections. The sections were dewaxed by xylene two times with 5 minutes each time, dehydrated with gradient ethanol (100%, 95%, 80%, and 75%) for 1 minute, respectively, and then washed under running water for 2 minutes. Next, the sections were stained with hematoxylin for 2 minutes, washed under running water for 10 seconds, and differentiated with 1% hydrochloric ethanol for 10 seconds. The sections were washed with distilled water for 1 minute, stained with eosin for 1 minute, washed with distilled water for 10 seconds, and then dehydrated with 95% ethanol two times and 100% ethanol two times with 1 minute each time. Finally, the sections were cleared with xylene, mounted with neutral balsam and observed at the microscope.

### 2.5 Immunohistochemistry

Samples were fixed with 4% formaldehyde, embedded with paraffin, cut into 4 μm serial sections and then placed in an incubator at 60°C for 1 hour. The sections were dewaxed with xylene, dehydrated with gradient ethanol, and incubated in 3% H_2_O_2_ (Sigma‐Aldrich Chemical Company, St. Louis, MO, USA) for 30 minutes at 37°C. Then, sections were washed with phosphate buffered saline (PBS), boiled in 0.01 mol/L citric acid buffer for 20 minutes at 95°C, washed with PBS after cooled to room temperature, and blocked with normal goat serum working solution for 10 minutes at 37°C. Subsequently, sections were added with 50 μL rabbit anti‐mouse FZD7 primary antibody (ab64636, 1:1000; Abcam, Cambridge, UK) at 4°C overnight and washed with PBS, added with the horse radish peroxidase (HRP)‐conjugated goat anti‐rabbit IgG second antibody (ab6789, 1:1000; Abcam) and incubated at 37°C for 30 minutes. The sections were rewashed with PBS three times with 3 minutes each time, and developed with diaminobenzidine (Sigma‐Aldrich Chemical Company). After that, the sections were re‐stained with hematoxylin (Bogoo Biotechnology, Shanghai, China) and then mounted with neutral resin. Substitution of PBS for the primary antibody was served as the negative control (NC), and the normal endometrial tissue was used for the positive control. The sections were observed and photographed under optical microscope (XSP‐36, Shenzhen Bo as of Optical Instrument Co., Ltd., Shenzhen, China) and five high power fields (HPF) (200×) of each section were randomly selected. A total of 100 endometrial epithelial cells were counted in each HPF, and the number of positive cells <10% indicated negative, positive cells ≥10% and <50% indicated positive, and positive cells>50% indicated strong positive.^16^

### 2.6 Dual‐luciferase reporter gene assay

The biological prediction website microRNA.org was performed to analyse the target gene of miR‐488, and the dual‐luciferase reporter gene assay was performed to prove whether FZD7 was the target gene of miR‐488. The 3'UTR gene fragment of amplified FZD7 was cloned, and the product of polymerase chain reaction (PCR) was cloned to the multiple clone sites of luciferase gene of pmirGLO (E1330, Promega, Madison, WI, USA) and named as pFZD7‐ wild type (Wt). Next, according to the predicted binding site of miR‐488 and its target gene, PCR site‐directed mutagenesis was conducted and pFZD7‐mutant type (Mut) vector was constructed. The pRL‐TK vector (E2241, Promega) expressing ranilla luciferase was used as internal reference to adjust the differences of cell number and efficiency of transfection. Lastly, miR‐488 mimic and corresponding NC were co‐transfected with luciferase reporter vector into epithelial cells of ectopic endometrium respectively. The activity of dual luciferase was measured according to the method provided by Promega.

### 2.7 Cell culture, grouping and transfection

Part of endometrial tissues of normal mice and mice with endometriosis was selected, washed with PBS two times, and inoculated in DMEM/F12 culture medium (GNM‐12500‐S, Shanghai Jingke chemical technology Co., Ltd., Shanghai, China), which contained 20% foetal bovine serum (FBS). After that, the tissues were incubated in 5% CO_2_ incubator at 37°C. The medium was changed with the appearance of a large number of desquamation of endothelial cells (every 3 days). The floating cells were collected. The medium was removed when cell confluence reached 80%‐90%, and then the cells were washed with PBS two times and treated with 0.25% trypsin. Subsequently, the concentration of cells was adjusted to 1.0 × 10^6^/mL by DMEM medium containing 10% calf serum, and then the cells were cultured in culture plate and culture dish in a certain amount. The purity of endometrial glandular epithelial cells was measured using antibody to keratin and then the cells were washed with PBS, added with 10 μL Alexa Fluor 488‐cytokeratin antibody (53‐9003‐82, Thermo Fisher Scientific, New York, USA) and 4ʹ,6‐diamidino‐2‐phenylindole (DAPI) (D21490, Thermo Fisher Scientific), observed and photographed under a fluorescence microscope (Olympus Optical Co., Ltd.). There are seven groups in this experiment: normal (cells of normal mice), blank (cells of endometriosis mice without transfection), NC (cells of endometriosis mice transfected with miR‐488 scrambled sequence), miR‐488 mimic (cells of endometriosis mice transfected with miR‐488 mimic), miR‐488 inhibitor (cells of endometriosis mice transfected with miR‐488 inhibitor), and si‐FZD7 (cells of endometriosis mice transfected with si‐FZD7), and miR‐488 inhibitor + si‐FZD7 groups (cells of endometriosis mice transfected with miR‐488 inhibitor and si‐FZD7). All the sequences were listed in Table 1. The plasmids used for transfection were all purchased from Sangon biotech Co., Ltd. (Shanghai, China). The cells in logarithmic growth phase that grew well were chosen with the concentration being adjusted to 1.2 × 10^5^ cells/mL and seeded in a 24‐well plate with 500 μL in each well until the cell confluency reached 50%.

**Table 1:** Sequences for transfection

Name	Sequence
miR‐488‐NC	5ʹ‐CUAUCAAUCGGCGGAUCCUAU‐3ʹ
miR‐488‐mimics	5ʹ‐UUGAAAGGCUAUUUCUUGGUC‐3ʹ
miR‐488‐inhibitors	5ʹ‐GACCAAGAAAUAGCCUUUCAA‐3ʹ
si‐FZD7	5ʹ‐UUAAAGUACAUCAGGCCGUUG‐3ʹ

Note

miR‐488, microRNA‐488; NC, negative control; si‐FZD7, silenced‐Frizzled7.

According to the instructions of lipofectamin2000 (11668019, Thermo Fisher Scientific, San Jose, CA, USA), 100 pmol miR‐488 mimic, miR‐488 inhibitor, si‐FZD7, miR‐488 inhibitor + si‐FZD7 and NC were diluted with 250 μL serum‐free medium Opti‐MEM (Gibco, Grand Island, NY, USA), and the ultimate concentration was 50 nmol/L. The mixture was incubated for 5 minutes at room temperature. Another 250 μL serum‐free medium Opti‐MEM was used to dilute 5 μL lipofectamine 2000, and the mixture was incubated for 5 minutes at room temperature. The two mixtures were mixed, incubated for 20 minutes at room temperature, and added into culture plate, which was shaken gently. The cells were incubated in CO_2_ incubator for 6‐8 hours at 37°C and then cultured in complete medium for 48 hours for following experiments.

### 2.8 TOP/FOPFlash reporter assay

Ectopic endometrial glandular epithelial cells were seeded into a 96‐well plate for 24 hours. According to the instructions of lipofectamin 2000 (11668019 Thermo Fisher Scientific), the transfection plasmids of different groups, TOPFlash or FOPFlash, and internal reference PRL‐TK plasmid (Madison, WI, USA) were added into each well. The plasmids were added into 100 μL L‐DMEM, mixed gently, and allowed to stand for 5 minutes at room temperature. A total of 0.5 μL lipofectamine 2000 and 100 μL L‐DMEM were mixed and allowed to stand for 5 minutes at room temperature. After the primary medium was washed away, the cells were washed with L‐DMEM and added with new mixture for transfection. Six hours after that, the cells were cultured in complete medium for 24‐48 hours and then the medium was discarded. Then the Dual‐Luciferase^®^ Reporter Assay System (E1910, Promega) was performed to determine the activity of firefly luciferase and ranilla luciferase of cells, and the ratio of firefly luciferase activity to ranilla luciferase activity was used to refer to the activation level of transcription factors of the Wnt/β‐catenin signalling pathway.^17^

### 2.9 Immunofluorescence assay

Cells were regularly detached, transfected, counted and spread in immunofluorescence chamber with 2 × 10^5^ cells in each well. When the cell confluency reached about 90%, the cells were washed with PBS three times on the ice. The cells were fixed by 4% paraformaldehyde (1 mL for each well), and then allowed to stand at room temperature for 15 minutes. After cells were washed with PBS three times, 0.3% Triton was used to perforate cells. Ten minutes later, the cells were washed with PBS three times, blocked with goat serum, and allowed to stand for 30 minutes. The cells were incubated with β‐catenin antibody (1:500, ab32572, Abcam) at 4°C overnight. The cells were washed with PBS three times, and incubated with Alexa Fluor 488 conjugated goat anti rabbit IgG (1:500, ab150077, Abcam) for 1 hour at room temperature avoiding exposure to light. After being washed three times, cells were added with DAPI to stain for 15 minutes avoiding exposure to light, and washed with PBS three times. Lastly, cells were mounted with fluorescence quenching agent and observed and photographed under a fluorescence microscope.

### 2.10 Reverse transcription quantitative PCR

Reverse transcription quantitative PCR (RT‐qPCR) was performed to detect the expression of FZD7 and downstream target genes in the Wnt signalling pathway. On the basis of related sequences published in the GeneBank, the primers of downstream target gene were designed by using Primer5.0 software and the homology analysis that carried out by BLAST (Table 2). Cells were collected after transfection, and the total RNA of cells was extracted by using the method of Trizol (Invitrogen Inc., Carlsbad, CA, USA) and reversely transcripted into cDNA according to the instructions of TaqMan MicroRNA Assays Reverse Transcription Primer (4427975, Applied Biosystems, Foster City, CA, USA). The cDNA was diluted to 50 ng/μL, 2 μL was addedeach time, and the reactive amplification system was 25 μL. The reaction conditions of reverse transcription were as follows: at 37°C for 30 minutes and at 85°C for 5 minutes. A total of 5 μL cDNA product was used as template for the PCR amplification. The PCR reaction system included: 5 μL product of reverse transcription product, 13 μL 2 × QuantiTect SYBR Green RT‐PCR Master Mix, 0.5 μL forward and reverse primers (10 μmol/μL each), respectively, and 6 μL DNAase‐free water. The reaction conditions were as follows: at 95°C for 5 minutes, 45 cycles of 95°C for 20 seconds, 60°C for 1 minute and 72°C for 30 seconds. Expressions of the genes were analysed, and the results of RT‐qPCR were analysed based on the 2‐ΔCt method. U6 was used as the internal reference for miR‐488, and β‐actin was used as the internal reference for FZD7, c‐Myc, β‐catenin, E‐cadherin and vimentin. Relative expression of the target gene was calculated based on the 2^−ΔΔCt^ method, and the formula was: ΔCt = Ct (target gene) − Ct (internal control), and ΔΔCt = ΔCt (experiment group) − ΔCt (control group). This experiment was repeated three times.

**Table 2:** Primer sequences for RT‐qPCR

Gene	Primer sequence (5’‐3’)
β‐actin	F: TTGCTGACAGGATGCAGAAG
	R: ACATCTGCTGGAAGGTGGAC
FZD7	F: GCCACACGAACCAAGAGGAC
	R: CGGGTGCGTACATAGAGCATAA
miR‐488	F: ACACTCCAGCTGGGTTGAAAGGCTGTTTC
	R: TGGTGTCGTGGAGTCG
U6	F: CTCGCTTCGGCAGCACA
	R: AACGCTTCACGAATTTGCGT
c‐Myc	F: GCTGGACACGCTGACGAAA
	R: TCTAGGCGAAGCAGCTCTATTT
β‐catenin	F: ATGGAGCCGGACAGAAAAGC
	R: TGGGAGGTGTCAACATCTTCTT
CyclinD1	F: GGCAGCCCCAACAACTTC
	R: TCCCGCCTGCCCGGTGG

Note

FZD7, Frizzled7; F, forward; miR‐488, microRNA‐488; RT‐qPCR, reverse transcription quantitative polymerase chain reaction; R, reverse.

### 2.11 Western blot analysis

The total protein of cells was extracted respectively, and the concentration of protein was determined by using BCA Protein Assay kit (20201ES76, Yeasen Company, Shanghai, China). A total of 20 μg protein were added in each well and separated with 8% sodium dodecyl sulphate polyacrylamide gel electrophoresis for 1 hour, and transferred onto a polyvinylidene fluoride membrane. The membrane was blocked with 5% skimmed milk, and then incubated with the following antibodies overnight at 4°C: β‐actin (ab8227, 1:1000), FZD7 (ab64636, 1:1000), c‐Myc (ab32072, 1:50), β‐catenin (ab16051, 1:4000, E‐cadherin (ab76055, 1:1000) and Vimentin (ab8978, 1:1000). All these antibodies were purchased from Abcam Inc. After washing with phosphate‐buffered saline with Tween 20 (PBST) three times, the membrane was incubated with the HRP‐labelled goat anti‐rabbit IgG secondary antibody (ab150077, 1:1000, Abcam Inc.) for 1 hour at room temperature, and then washed with PBST three times. Subsequently, the membrane was exposed in darkroom by using Electrochemiluminescence (ECL) solution (ECL808‐25, Biomiga, San Diego, CA, USA), and then pressed. The relative expression of target protein was presented by the ratio of the grey level of target protein band to the β‐actin band.

### 2.12 3‐[4,5‐dimethylthiazol‐2‐yl]‐2,5 diphenyl tetrazolium bromide (MTT) assay

Cells in logarithmic growth phase were prepared into cell suspension with a density of 2.5 × 10^5^ cells/ml by using RPMI 1640 medium containing 10% FBS. Diluted cell suspension was seeded in a 96‐well plate, and 8 wells were prepared for each group with 100 μL in each well. The cells were incubated in a 5% CO_2_ incubator at 37°Cfor 24, 48 and 72 hours respectively. Then the culture plate was removed and 10 μL MTT solution (Sigma Corporation of America, New York, USA) (5 mg/mL) was added into each well to incubate cells for 4 hours. The optical density of each well was determined by automatic enzyme meter (BioRad, Richmond, California, USA) at 490 nm. This experiment was repeated three times.

### 2.13 Scratch test

After 48‐hour transfection, cells were seeded into a 6‐well plate. When cells adhered to wall, they were cultured by serum‐free DMEM. When cell confluence reached 90%‐100%, a 10 μL‐pipette was used to perpendicularly and slowly scratch on the bottom of the culture plate, and 4‐5 wounds with the same width were created in each well. The cells were washed with PBS three times to remove scratched cells and then incubated in incubator. After 0 and 24 hours of scratch, inverted microscope was performed to observe the migration distance of cells, and cells were photographed under several randomly‐selected fields. This experiment was repeated three times.

### 2.14 Transwell assay

Transwell chambers (Corning, New York, USA) with a 24‐well plate and 8 μm‐size wells were used in this experiment. A total of 200 μL cells were seeded in apical chamber, 300 μL complete medium (Invitrogen Corp.) with 10% FBS was added into the basolateral chamber as chemotactic factor. Then the Transwell chambers were incubated in 5% CO_2_ incubator at 37°C. Before 48 hours of immobilization and staining, 50 μL Matrigel (Sigma Corporation of America) was spread in Transwell chambers to package the filter membrane. Stained cells were counted by using an inverted microscope. Cells were counted under randomly‐selected fields to calculate the mean value. The method above was applied to assess the invasion of tumour cells.

### 2.15 Statistical analysis

Statistical analyses were conducted by using spss 19.0 (IBM, Armonk, NY, USA). Measurement data were expressed as mean ± SD. Comparison between two groups was performed by *t* test. Comparison among multiple groups was conducted by one‐way anova. Results were expressed as percentage and analysed using chi‐square test. *P* < 0.05 was considered significantly different.

## 3 RESULTS

### 3.1 FZD7 is identified as an upregulated gene in endometriosis

**Figure 1:**
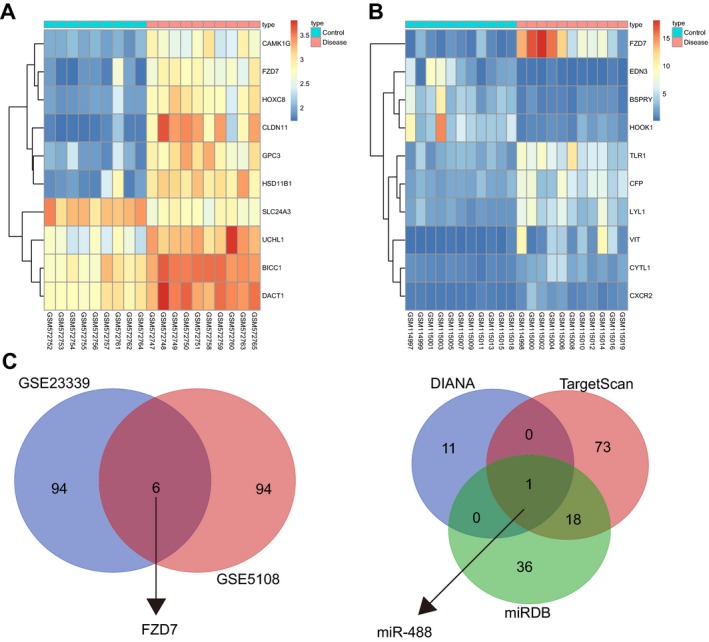
FZD7 is identified as an up‐regulated gene in endometriosis, based on differential analysis using the GSE23339 and GSE5108 chips. (A,B) heat map for DEGs in the GSE23339 and GSE5108 chips; the abscissa indicates the sample number; the ordinate indicates the gene name; the upper dendrogram represents the sample type clustering; the histogram of the upper right represents a color gradation: red color indicates a high expression and green color indicates a low expression; each square in the graph represents the expression of one gene in one sample; the left dendrogram represents gene expression clustering; (C) Venn map for the top 100 DEGs in the GSE23339 and GSE5108 chips; (D) Venn analysis that predicates the miRNA regulating FZD7; blue color shows the prediction result from the DIANA database, the red color shows the prediction result from the TargetScan database and the green color shows the prediction result from the miRDB database. FZD7, Frizzled‐7; DEGs, differentially expressed genes

We downloaded the gene expression datasets GSE5108 and GSE23339 from the GEO database. In the GSE5108 chip, we screened 1856 DEGs related to endometriosis and in the GSE23339 chip, we screened 1372 DEGs. According to the previous literatures, FZD7 was a new prognostic indicator, which promoted tumour metastasis through the WNT and epithelial‐to‐mesenchymal transition (EMT) signalling pathways in oesophageal squamous cell carcinoma.^18^ FZD7 K‐O inhibited cell growth and metastasis and promoted chemosensitivity of oesophageal squamous cell carcinoma cells by inhibiting the Wnt signal transduction. Moreover, FZD7 regulated bone marrow mesenchymal stem cell‐mediated chronic myeloid leukaemia cell protection.^19^, ^20^ Therefore, FZD7 played a carcinogenic role in many human cancers. However, the exact function of FZD7 in human endometriosis was unclear; thus FZD7 gene became a candidate gene that we were interested in. We aimed to predict the function and clinical significance of FZD7 in endometriosis. Moreover, FZD7 ranked highest among the most DEGs. Figure 1A is a heat map of DEGs in the GSE23339 chip and Figure 1B is a heat map of DEGs in the GSE5108 chip. From Figure 1A,B, it was found that the FZD7 gene was upregulated in endometriosis. In order to study the upstream of the DEG, FZD7, we predicted the miRNAs capable of regulating FZD7 through the bioinformatics website, combining with the use of a Venn map. The result showed that only one gene was co‐expressed, namely, miR‐488 (Figure 1C,D).

### 3.2 The endometrial tissues appear lesions in mice with endometriosis

**Figure 2:**
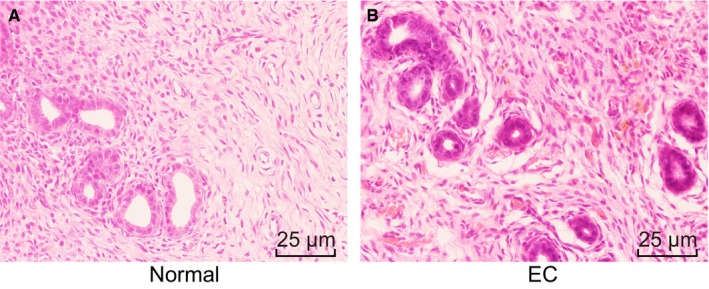
Lesions are appeared in the endometrial tissues of mice with endometriosis (×400). (A) the normal endometrial tissue; (B) the ectopic endometrial tissue

Initially, hematoxylin eosin (HE) staining was performed to observe the detailed differences in tissues of ectopic endometrium. The results indicated that the nidus of most of mice with endometriosis appeared typical endometrioid tissues, which had similar morphological structure as normal endometrium (Figure 2). Meanwhile, the nidus appeared atypical endometrial tissues, and then mainly appeared fibrous connective tissue, accompanied by endometrial stroma and glandular epithelium with increasing number of weeks. Therefore, mice with endometriosis had pathological changes in endometrial tissues.

### 3.3 High expressions of FZD7 and Wnt pathway‐related factors and low miR‐488 expression are found in ectopic endometrial tissue of mice

**Figure 3:**
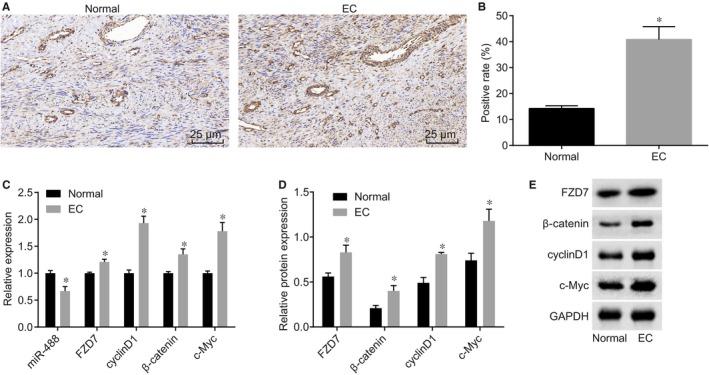
Elevated mRNA and protein expression of FZD7 and Wnt pathway‐related factors, and reduced miR‐488 expression are found in ectopic endometrial tissue. (A) the positive expression of FZD7 in the ectopic endometrial tissue and normal endometrial tissue (×400); (B) the positive rate of FZD7 protein in the ectopic endometrial tissue and normal endometrial tissue; (C) comparison of mRNA expression of related factors in the ectopic endometrial tissue and normal endometrial tissue; (D) the protein expression of related factors in the ectopic endometrial tissue and normal endometrial tissue; (E) protein bands of FZD7, β‐catenin, cyclinD1 and c‐Myc in ectopic endometrial tissues and normal endometrial tissue examined by western blot analysis; *, *P < *0.05 compared with the normal endometrial tissue (n = 30, comparison between two groups was performed by *t* test); miR‐488, microRNA‐488; FZD7, Frizzled‐7.

Immunohistochemistry, RT‐qPCR and western blot analysis were employed to examine the expressions of miR‐488, FZD7 and Wnt pathway‐related factors in endometrial tissues. As shown in Figure 3A, FZD7 positive protein was brown yellow and located in cytoplasm and cell membrane. The positive expression of FZD7 protein in endometrial tissue of normal mice was 14.25%, while that of mice with endometriosis was 40.87% (Figure 3B), suggesting a significant increase of the expression of FZD7 in ectopic endometrial tissue of mice with endometriosis (*P* < 0.05). Besides, the results of RT‐qPCR and western blot analysis (Figure 3C‐E) indicated that compared with the normal endometrial tissue, ectopic endometrial tissue showed higher mRNA and protein expressions of FZD7, β‐catenin, cyclinD1 and c‐Myc but lower miR‐488 expression (all *P < *0.05). Therefore, it can be concluded that ectopic endometrial tissues of mice had higher mRNA and protein expressions of FZD7 and Wnt pathway‐related factors but lower miR‐488 expression.

### 3.4 FZD7 is a target gene of miR‐488

**Figure 4:**
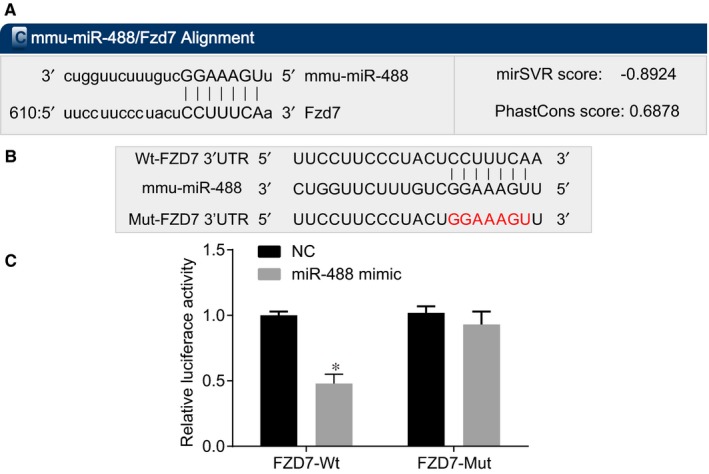
FZD7 is a target gene of miR‐488. (A) the binding site of FZD7 and miR‐488 predicted by bioinformatics website; (B) the sequences of FZD7‐Wt and FZD7‐Mut; (C) the luciferase activity of FZD7‐Wt and FZD7‐Mut in the NC and miR‐488 mimic groups; *, *P < *0.05 compared with the NC group (n = 30, comparison of two groups was performed by independent sample *t* test); miR‐488, microRNA‐488; FZD7, Frizzled‐7; NC, negative control; Wt, wild type; Mut, mutant type.

According to the online bioinformation analysis website microRNA.org, the target binding site of FZD7 and miR‐488 existed (Figure 4A) and the target sequences of FZD7‐wild type (Wt) and FZD7‐Mut are shown in Figure 4B. Besides, the results of dual‐luciferase reporter gene assay indicated that compared with the NC group, the co‐transfection of miR‐488 mimic and Wt‐miR‐488/FZD7 group had lower luciferase activity (*P < *0.05) (Figure 4B), while that of the Mut‐miR‐488/FZD7 had no significant difference (*P* > 0.05). These results suggested that miR‐488 could bind to FZD7 mRNA specifically.

### 3.5 MiR‐448 inhibits the activation of Wnt signalling pathway via suppression of the FZD7

**Figure 5:**
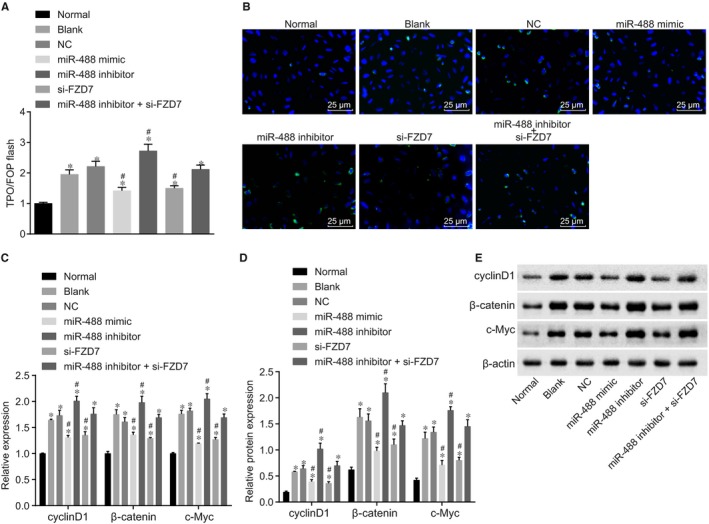
Up‐regulated miR‐488 inhibits the activation of Wnt signaling pathway via inhibition of FZD7. (A) the differences in TCF/LEF transcription activities of cells in seven groups detected by TOP/FOPFlash; (B) the β‐catenin expression in nucleus detected by immunofluorescence assay (×400); (C) the expression of genes associated with Wnt signaling pathway in seven groups detected by RT‐qPCR; (D,E) the relative protein expression and protein bands of genes associated with Wnt signaling pathway in seven groups detected by western blot analysis; *, *P < *0.05 compared with the normal group; #, *P < *0.05 compared with the blank and NC groups, (comparison of multiple groups was performed by anova); miR‐488, microRNA‐488; FZD7, Frizzled‐7; RT‐qPCR, reverse transcription quantitative polymerase chain reaction; NC, negative control.

Luciferase reporter gene of firefly was found in the TOP‐Flash plasmid, three repeated TCF binding sequences in the upstream of luciferase promoter could control the expression of downstream luciferase according to the activity of β‐catenin. The TCF binding sequences in TOPFlash plasmid were mutant, other sequences are consistent with FOPFlash and not affected by the activity of β‐catenin. So TOP/FOPFlash was usually used as an index to detect the activation of Wnt/β‐catenin signalling pathway. The key point of the activation of Wnt/β‐catenin signalling pathway was that the β‐catenin accumulated and entered the nucleus, and combined with transcription factor TCF/LEF to co‐control the gene expression. To further explore the effect of miR‐488 on the Wnt signalling pathway by regulating FZD7, the endometrial glandular epithelial cells were extracted from the endometrial glandular epithelial tissues from normal and endometriosis mice and then identified, and the results (Supporting Information Figure S1) showed that cells under a microscope presented obvious epithelioid cell morphology and the positive cells of cytokeratin staining accounted for 80%. The endometrial glandular epithelial cells of normal and endometriosis mice were transfected, respectively and then following experiment was conducted. The results of TOPFlash indicated that the activation of TOPFlash was increased in the miR‐488 inhibitor group but was decreased by over‐expressed miR‐488, suggesting that over‐expressed miR‐448 inhibited the activation of Wnt/β‐catenin signalling pathway (Figure 5A). Immunofluorescence staining was performed for the further analysis of β‐catenin expression in nucleus. As shown in Figure 5B, the miR‐488 mimic and si‐FZD7 groups showed lower fluorescent expression of β‐catenin protein and significantly lower expression in nucleus. Contrarily, the miR‐488 inhibitor group exhibited higher fluorescent expression of β‐catenin protein and the expression transferred to nucleus. There was no significant difference of fluorescent expression among the miR‐488 inhibitor + si‐FZD7, blank and NC groups. RT‐qPCR and western blot analysis were applied to examine the expressions of Wnt/β‐catenin signalling pathway‐related factors, and the results (Figure 5C‐5E) showed that the expressions of cyclinD1, β‐catenin and c‐myc in the other groups were higher than that in the normal group (all *P* < 0.05). There was no significant difference of the expression of cyclinD1, β‐catenin and c‐myc between the blank and NC groups (*P* > 0.05). Compared with the blank and NC groups, the miR‐488 mimic and si‐FZD7 groups showed lower mRNA and protein expressions of cyclinD1, β‐catenin and c‐myc, while the miR‐488 inhibitor group showed obviously higher mRNA and protein expressions of these genes (*P* < 0.05). There was no significant difference of the expressions in the miR‐488 inhibitor + si‐FZD7 group (*P* > 0.05). Therefore, it can be concluded that elevated miR‐488 inhibits the FZD7 expression and the activation of the Wnt signalling pathway.

### 3.6 Overexpression of miR‐488 inhibits the proliferation, migration and invasion of ectopic endometrium cells

**Figure 6:**
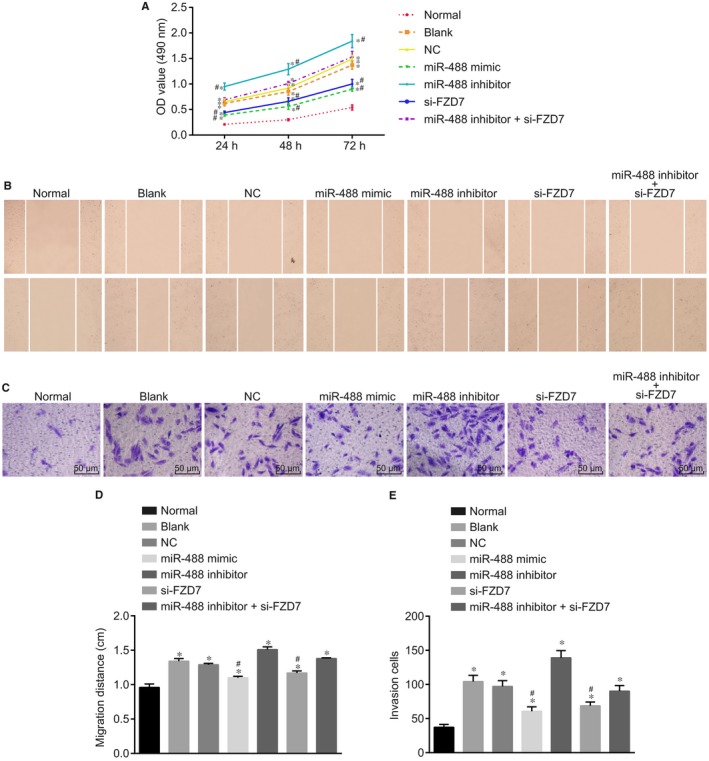
Up‐regulated miR‐488 and down‐regulated FZD7 inhibit the proliferation, migration, and invasion of endometrial glandular epithelial cells. (A) the differences of viability of cells in seven groups; (B,D) migration of cells in seven groups, overexpressed miR‐488 inhibited cell migration (×200); (C,E) invasion of cells in seven groups (×200), cell invasion was suppressed after transfection of up‐regulated miR‐488; *, *P < *0.05 compared with the normal group; #, *P < *0.05 compared with the blank and NC groups, (comparison of multiple groups was performed by anova); miR‐488, microRNA‐488; FZD7, Frizzled‐7; NC, negative control

MTT assay, scratch test and Transwell assay were performed to detect the proliferation, migration and invasion of ectopic endometrium cells. According to the results of MTT assay shown in Figure 6A, the proliferation rate of cells in the other groups was higher than that in the normal group. There was no significant difference in cell proliferation between the blank and NC groups (*P* > 0.05). Compared with the blank and NC groups, the miR‐488 inhibitor group had obviously higher activity of cell proliferation, while the miR‐488 mimic and si‐FZD7 groups had lower activity of cell proliferation (all *P* < 0.05). There was no significant difference in the miR‐488 inhibitor + si‐FZD7 group (*P* > 0.05). These results suggested that overexpressed miR‐488 and silenced FZD7 inhibited the proliferation of ectopic endometrium cells. The results of scratch test and Transwell assay (Figure 6C‐6E) indicated that the migration and invasion of cells in the other groups were higher than in the normal group. There was no significant difference in cell migration and invasion between the blank and NC groups (*P* > 0.05). Compared with the blank and NC groups, the miR‐488 mimic and si‐FZD7 groups had decreasing migration and invasion (all *P* < 0.05), the miR‐488 inhibitor group had obviously enhanced migration and invasion (*P* < 0.05). There was no significant difference in the miR‐488 inhibitor + si‐FZD7 group when compared with the blank and NC groups (*P* > 0.05). These results suggested that overexpressed miR‐488 and FZD7 gene silencing suppressed the proliferation, migration and invasion of endometrial glandular epithelial cells.

## 4 DISCUSSION

It is well known that endometriosis is a chronic disease that has affected about 5‐10% of reproductive‐age women, and is defined as the abnormal presence of endometrial glands and stroma outside the uterus, primarily in the pelvic cavity.^2^ Recent studies have identified miRNAs as key regulators of gene expression, and dysregulated miRNA expression may be involved in the endometriosis pathogenesis.^9^, ^21^, ^22^ In this study, we aimed to find out the mechanism that miR‐488 affected endometrial glandular epithelial cells in mice with artificial endometriosis. Consequently, this study demonstrated that up‐regulated miR‐488 suppressed Wnt signalling pathway by inhibiting FZD7, thus inhibited the proliferation, migration and invasion of endometrial glandular epithelial cells.

In this study, we observed that miR‐488 expression was decreased while FZD7 was increased in artificial ectopic endometrial tissue when compared with normal endometrial tissue, which indicated miR‐488 and FZD7 had important roles in the endometriosis initiation and progression. A previous study has revealed that the expression of miR‐488 was reduced in ovarian cancer tissues, indicating that it may play an important role in regulating mitochondrial function and chemoresistance in ovarian cancer.^10^ In addition, it has been previously shown that miR‐488 was down‐regulated in cancer tissues, and the expression of miR‐488 was obviously lower in high tumour‐node‐metastasis (TNM) grade tissues than that in low TNM grade tissues^23^ According to another study, highly expressed FZD7 was found in gastric cancer, hepatocellular cancer as well as oesophageal cancer.^24^ Furthermore, one previous study demonstrated that intensive inhibition of FZD7 could be used as a reasonable and promising new tumour treatment method.^25^ Thus, we could conclude the important role played by miR‐488 and FZD7 in endometriosis. In the subsequent experiments, we demonstrated the function of miR‐488 on endometrial cells with the involvement of the Wnt signalling pathway and FZD7.

After cell transfection, we also observed up‐regulated miR‐488 inhibited proliferation, migration and invasion of ectopic endometrium cells through suppression of the Wnt signalling pathway. Besides, based on the target prediction program and the luciferase activity determination, we found that FZD7 is a putative target gene of and negatively governed by miR‐488. Song et  al demonstrated that miR‐488 expression was decreased significantly in osteoarthritis chondrocytes, while overexpressed miR‐488 promoted chondrocyte differentiation and cartilage development through MMP‐13 by binding to ZIP‐8.^26^ The result of a recent study demonstrated that miR‐488 expression was inhibited in gastric cancer, compared with tissues without tumour, and the proliferation and migration of gastric cancer cell were suppressed by the up‐regulated miR‐488.^27^ In the study of Dengdi Hu et  al, the unusually low expression of miR‐488 was also observed in hepatocellular carcinoma tissues, and the important role of miR‐488 in suppressing proliferation, colony formation, cell invasion and EMT process has been reported.^11^ FZD7, which is widely known as the most commonly reporter of Wnt, has been identified as a target for cancer therapy, since it can play an important role in controlling endothelial cell proliferation through the inhibition of the Wnt/β‐catenin signalling regulators.^28^ According to the previous studies, the overexpressed FZD7 can be found in multiple solid cancers and implicated in the carcinogenesis and tumour progression, thus leads to the shorter survival of colorectal and gastric cancer patients.^12^ In accord with some previous studies, our study demonstrated that the inhibition of FZD7 was associated with blocked Wnt signalling pathway. It is well documented that Wnt/β‐catenin signalling plays an important role in promoting oncogenic activities of cell proliferation, as well as cell invasion in glioma, and inhibiting apoptosis of cells.^13^ The study conducted by Ana M. Sanchez et  al have revealed that the Wnt/β‐catenin signalling may play a crucial role in suppressing cell apoptosis.^29^ Hence, we suggest that FZD7 may regulate Wnt signalling pathway, which is controlled by miR‐488.

**Figure 7:**
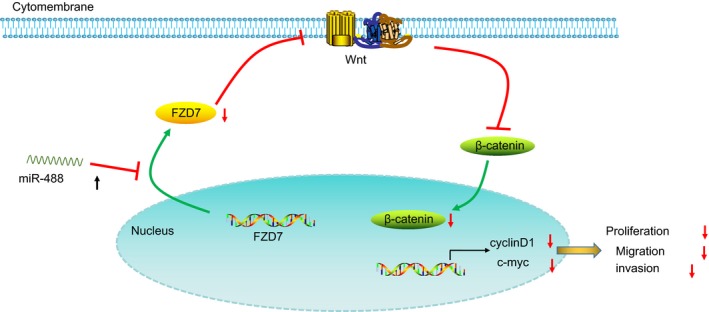
The mechanism of miR‐488 involved in endometriosis; microRNA‐488 exerted inhibitory effects on endometrial glandular epithelial cell proliferation, migration, and invasion of mice in endometriosis via the Wnt signaling pathways by inhibiting FZD7

In summary, according to what has been concluded in the present study, we suggests that overexpressed miR‐488 inhibits the Wnt signalling pathway by targeting FZD7, thus suppressing proliferation, migration, invasion of endometrial cells and act as a protective role in mice with artificial endometriosis (Figure 7). Given that low level of miR‐488 is confirmed in relation to mouse endometriosis progression, our discovery outlines a potential molecular target for endometriosis treatment.

## CONFLICT OF INTEREST

The authors report no conflicts of interest in this work.
